# Serum metabolomics-based diagnostic biomarkers for colorectal cancer: insights and multi-omics validation

**DOI:** 10.3389/fendo.2025.1663938

**Published:** 2025-10-27

**Authors:** Taorui Wang, Yuanxu Gao, Zhihai Liu, Peng Du, Shengjun Tang, Zijun Lai, Gen Li

**Affiliations:** ^1^ Faculty of Medicine, Macau University of Science and Technology, Taipa, Macao SAR, China; ^2^ Institute for AI in Medicine, Macau University of Science and Technology, Taipa, Macao SAR, China; ^3^ Genetic Testing Center, Guangzhou Medical University, Guangzhou, China; ^4^ Guangzhou Women and Children’s Medical Center, Guangzhou Medical University, Guangzhou, China; ^5^ National Clinical Research Center for Ocular Diseases, Eye Hospital, Wenzhou Medical University, Wenzhou, China

**Keywords:** colorectal cancer, metabolomics, biomarker, machine learning, diagnostic model

## Abstract

**Background:**

Colorectal cancer (CRC) remains one of the leading causes of cancer-related mortality worldwide, primarily due to delayed diagnosis. There is an urgent need for sensitive, noninvasive biomarkers that can facilitate early detection and improve clinical outcomes.

**Methods:**

In this study, we performed untargeted metabolomic profiling of serum samples from 715 participants (248 CRC patients and 467 noncancer controls, NCC) using liquid chromatography-mass spectrometry (LC-MS). Differential metabolites were identified through statistical filtering and multivariate analysis, followed by pathway enrichment to elucidate biologically relevant dysregulations. Subsequently, machine learning algorithms, including Support Vector Machine (SVM), Random Forest (RF), eXtreme Gradient Boosting (XGBoost), and Logistic Regression (LR), were applied to construct predictive models. As a complementary approach, we also profiled cfDNA methylation patterns in a subset of samples and developed a multi-omics classifier integrating metabolite and epigenetic features.

**Results:**

We identified 26 CRC-associated serum metabolites, many of which mapped to dysregulated pathways such as primary bile acid biosynthesis and taurine/hypotaurine metabolism, suggesting active reprogramming of host-microbiota metabolic axes in CRC pathogenesis. A metabolomics-based diagnostic model built using ten selected metabolites demonstrated excellent discriminatory performance, achieving area under the receiver operaring characteristic curve (AUROC) of 0.96-0.97 and accuracies up to 92.5% across multiple machine learning methods. Integration of cell-free DNA (cfDNA) methylation markers yielded a multi-omics model with slightly enhanced performance (AUROC=0.98), but the gain over the metabolomics-only model was modest.

**Conclusion:**

This study reveals distinct serum metabolic signatures and pathway disruptions in CRC patients and presents a high-performance, minimally invasive diagnostic model based solely on metabolomics data. While the integration of methylation features offers incremental benefit, metabolomics remains the dominant predictor, underscoring its potential as a standalone platform for early CRC screening and precision medicine.

## Introduction

Colorectal cancer (CRC) ranks as the third most commonly diagnosed cancer and the second leading cause of cancer-related deaths globally, accounting for 9.6% of all cancer cases and 9.3% of cancer-related deaths worldwide (1). In China, the latest annual cancer report published by the National Cancer Centre indicates that CRC is the second most frequently diagnosed cancer and the fourth leading cause of cancer death in 2022. Trends in incidence and mortality reveal gender disparities: among women, both incidence and mortality rates are declining, whereas among men, these rates are on the rise. Overall, CRC poses a significant threat to public health and imposes a substantial disease burden, both in China and globally.

Traditional methods for early cancer screening include medical imaging, genetic testing, and tissue biopsy. These approaches are often limited by low sensitivity, high cost, and invasiveness. In addition, the United States Preventive Services Task Force (USPSTF) recommends several screening tests for colorectal cancer, including the fecal occult blood test (annually), multitarget fecal DNA test (every 3 years), colonoscopy (every 10 years), and computed tomographic colonography (every 5 years) (2, 3), which leads to time-consuming and labor-intensive screening, resulting in low patient compliance. Therefore, identifying more sensitive and specific biomarkers and developing noninvasive, easy-to-operate screening methods capable of simultaneously screening for CRC is essential. These advancements can expand tumor screening coverage, facilitate early diagnosis and treatment, and help curb the increasing cancer burden.

In recent years, metabolomics has emerged as a promising approach for cancer screening, including colorectal cancer (CRC) (4, 5). This field involves the systematic study of small-molecule metabolites in biological fluids, cells, and tissues. Research into its potential applications for discovering cancer biomarkers is rapidly expanding (6–9). Several studies have reported metabolite-based signatures associated with CRC (10, 11), however, most suffer from small sample sizes, lack of independent validation, or limited integration of advanced computational modeling (12–14). Moreover, while multi-omics approaches, such as integrating metabolomics with epigenetic profiling, hold promise for enhancing diagnostic accuracy, the relative contribution of each modality remains underexplored. In particular, the standalone diagnostic power of serum metabolomics has yet to be fully delineated in large, clinic-based diagnostic cohorts using rigorous machine learning frameworks.

In this study, we aimed to identify serum metabolite signatures associated with CRC and construct robust diagnostic models using multiple machine learning algorithms. We further interpreted the biological relevance of the dysregulated metabolites through pathway analysis, with a particular focus on host-microbiota metabolic interactions. Finally, we incorporated cfDNA methylation markers in a subset of samples to evaluate the potential benefit of a multi-omics strategy, while maintaining serum metabolomics as the central diagnostic platform.

## Materials and methods

### Clinical samples

A total of 715 serum samples were collected from the Zhuhai People's Hospital (the First Affiliated Hospital of Macau University of Science and Technology) between 2020 and 2023, including 248 patients diagnosed with CRC and 467 noncancer controls (NCCs) (**Table 1**). CRC diagnoses were confirmed histopathologically. Control participants underwent routine colonoscopy and were confirmed to be free of malignant or precancerous lesions. All participants provided informed consent, and the study protocol was approved by the institutional ethics committee.

**Table 1:** Baseline characteristics of the study participants.

Characteristic	ALL	CRC	NCC	*P* value
	N=715	N=248	N=467	
Gender, No. (%)				0.505
female	325 (45.5)	108 (43.5)	217 (46.5)	
male	390 (54.5)	140 (56.5)	250 (53.5)	
Age, Mean (SD)	59.5 (10.6)	60.5 (10.9)	58.9 (10.4)	0.065
BMI (kg/m^2^)	22.8 (2.21)	23.0 (2.30)	22.7 (2.16)	0.077
TNM stage	NA	I: 50	NA	
		II: 71		
		III: 82		
		IV: 33		
		No record: 12		

Blood samples were collected by venipuncture, and the patient was required to fast for at least 8 hours but not more than 16 hours, preferably 12–14 hours. The serum was separated within 2h and centrifuged at 3000 rpm for 10min at room temperature. The supernatant was transferred to a centrifuge tube and centrifuged again at 14,000 rpm for 10min at 4 °C. Serum was obtained from the supernatant, and the serum samples were frozen and stored at −80 °C until sample processing.

### Sample preparation for metabolomics

All the serum samples stored at −80 °C were first thawed on ice before preparation. After the samples were vortexed for 30 s, a 10 μL aliquot of serum from each sample (including all patients with tumors and healthy controls) was mixed thoroughly for quality control (QC). Both the serum and QC samples were extracted at a volume ratio of 100 μL, and 400 μL of MeOH was added to each sample to initiate protein precipitation and metabolite extraction. After being vortexed for 30 s, the mixture was centrifuged at 14,000 rpm for 10min at 4°C. Two hundred microliters of the supernatant were transferred to new Eppendorf (EP) tubes, and the samples were subsequently dried on a speed vac for 150min at 37°C and stored at −80°C. Before UPLC-MS analysis, the dried samples were redissolved in 50 μL of ultrapure water. The samples were vortexed for 30 s and subsequently sonicated in a water bath for 30 s, followed by centrifugation at 14,000 rpm for 10min at 4°C. Finally, 20 μL of the supernatant was collected and analyzed immediately. The pooled QC sample was injected five times at the beginning of the analysis to ensure system equilibrium, after which it was injected every ten samples during serum sample detection to further monitor system stability.

### UPLC-MS experiments

Untargeted LC-MS profile analysis of polar metabolites was performed on a UPLC system (ACQUITY UPLC I-Class system, Waters Corp.) coupled with tandem ESI–QTOF mass spectrometry (Synapt G2-Si, Waters Corp.). A 2 μL sample was injected into the chromatograph and separated on an ACQUITY UPLC HSS T3 1.8 μm, 2.1× 100mm i.d. column (Waters Corp.). The column temperature was controlled at 30°C. Mobile phase A was H_2_O containing 0.1% formic acid, and mobile phase B was 0.1% formic acid in CAN. During the entire analysis, the autosampler temperature was maintained at 4°C to avoid sample degradation. Two injections were performed for each sample to collect positive and negative data in full scan mode with a mass range of 50 to m/z at a resolution of 10,000. The electrospray ionization (EI) capillary voltages and cone voltages were set as 2.0 kV and 20V, respectively. The source temperature was 100°C, and the desolvation temperature was 200°C. The desolvation gas flow rate was 500 L/h.

### Metabolic data processing and statistical analysis

The raw MS data were converted to mzXML format using MSConvert in the ProteoWizard software package (v3.0.23089) (15) and processed using the R-based XCMS package (16) for peak extraction, peak optimization, retention time alignment, feature formation, feature grouping, aggregation, spectrum extraction, spectrum aggregation, compound identification, and quantitative data generation. The following parameters were used: peak width = c (5, 20), noise = 1000, snthresh = 3, ppm = 20, binSize = 6, minFraction = 0.4, bw =20. The compound annotations of the metabolites were matched with the Human Metabolome Database (HMDB) (17) and the Kyoto Encyclopedia of Genes and Genomes (KEGG) (18) database by metID (19). The parameters used were ms1.matchh.ppm = 15, rt. match.tol = 30, threads = 30, column = rp (20). The ‘metid’ package was used for metabolite identification based on public databases.Candidates with the highest spectral similarity to fragmentation patterns from databases (HMDB and KEGG) were prioritezed. Candidates present in two databases were given higher priority, reducing reliance on single-database matches. Annotations consistent with expected adduct patterns (e.g., [M+H]+, [M+Na]+) were retained, while ambiguous matches were flagged for manual review. Redundant annotated metabolites are removed based on Level and score, retaining compounds with the smallest level and highest similarity score (SS). Before statistical analysis, the serum metabolomics peak intensity data were log2-transformed. QC-based robust LOESS signal correction (QC-RLSC) (21) was utilized for data normalization to correct for systematic bias, and features with relative standard deviations (RSDs) of more than 35% in the QC sample were filtered out.

After normalization, the data were analyzed using the R package ropls for multivariate statistical analysis and modeling, including principal component analysis (PCA) and orthogonal partial least squares discriminant analysis (OPLS-DA). The quality of the model was tested by 7-fold cross-validation, and 20 permutation tests further tested the validity of the model. R2 and Q2 represent the explanatory and predictive abilities of the model, respectively. The variable importance for projection (VIP) value denotes the contribution of the feature peaks to the classification. The Wilcoxon rank-sum test was used to compare the metabolite levels between patients with tumors and healthy controls, and the false discovery rate (FDR) was used for *p*-value correction. FDR < 0.05 and VIP > 1 were used to screen significantly changed metabolites. Spearman’s correlation analysis was performed to analyze the associations between metabolites. Enrichment pathway analysis was performed using the web-based MetaboAnalyst 6.0 software.

### Screening of endogenous metabolites

Classification was strictly based on annotations from two authoritative databases: HNDB and MedChemExpress. HMDB’s “Biological Role” and “Origin” fields served as key references, with metabolites classified as endogenous if annotated as “endogenous” (i.e., produced by human cells or resident microbiota) and detected in “blood” or “plasma” according to the database’s tissue localization data. For metabolites with limited annotation in HMDB, MCE’s “Compound Type” classification was utilized, excluding those explicitly labeled as “exogenous” (e.g., dietary phytochemicals, pharmaceutical drugs, or environmental pollutants). Additionally, literature evidence was consulted to identify the main sources of metabolites.

### Methylation experiment

To explore the potential added value of integrating epigenetic information, cfDNA methylation profiling was conducted on a subset of 197 samples (68 CRC, 129 NCC). Targeted bisulfite sequencing was used to detect CRC-associated hypermethylated loci. cfDNA was isolated from the serum using a Magen cfDNA extraction kit following the manufacturer’s instructions and then ligated to a methylation adaptor using an NEBNext Ultra II DNA library Prep Kit for Illumina from NEB. Adaptor-ligated cfDNA was 12-to-1 mixed and hybridized with customized probes (Integrated DNA Technologies) using an xGen hybridization capture DNA libraries Kit (Integrated DNA Technologies). Hybridized mixture samples were eluted using the reagents and steps of the ‘‘Second Elution’’ part of the TruSeq Methyl Capture EPIC Library Prep Kit (FC-151-1003, Illumina) and then bisulfite converted using an EZ-96 DNA Methylation-Lightning Mag Prep Kit (D5047, ZYMO RESEARCH). Bisulfate-converted samples were amplified using the reagents and steps of the ‘‘Amplify Enriched Library’’ section of the TruSeq Methyl Capture EPIC Library Prep Kit (FC-151-1003, Illumina). The concentration of the prepared libraries was determined with a Qubit 2.0 fluorometer (Invitrogen, Life Technologies), and the library quality was assessed by capillary electrophoresis (Qsep100, Bioptic). The qualified libraries were sequenced on the Illumina Nova-seq platform (Illumina).

### Methylation data processing and statistical analysis

Features with more than 50% missing values and samples with more than 20% missing values were filtered out, and then the median of abundance was used to fill in the missing values. After data filtering and normalization, the limma function performed a difference analysis on the normalized data. Benjamini-Hochberg correction was performed to reduce the bias caused by multiple tests, and the probes with *p*-values < 0.05 were filtered. Probes with Δβ > 0.1 or Δβ < -0.1 were considered hypermethylated or hypomethylated, respectively. The genes corresponding to the differential probes were annotated using the org.Hs.eg.db R package with UCSC.hg19 as the reference genome file.

### Metabolomic and methylation features integration

Feature-level integration concatenated filtered metabolomic and methylation features into a unified matrix. To ensure compatibility between the two omics layers, composite data were normalized using M-value transformation for better interpretability in linear models. The datasets were scaled to zero mean and unit variance to prevent bias toward higher-magnitude features. The final integrated feature set was then input into the downstream diagnostic model.

### Construction of machine learning diagnostic models

In our study, all participants were randomly stratified and sampled into training and test datasets at a ratio of 7:3. Feature selection and model construction were performed on the training set, and hyperparameters were optimized through cross-validation. Subsequently, validation was carried out on the test set.

Features were selected using the least absolute shrinkage and selection operator (LASSO) and random forest (RF) algorithms. We performed LASSO regression on the training dataset to select the features with nonzero coefficients as a small number of features capable of identifying patients with tumors based on the average misclassification error of 10 random cross-validations (according to default settings). Then, the RF algorithm was used to calculate the relation importance of individual differential features, and the differential features were rounded off by performing 5-fold cross-validation five times (according to default settings). Finally, a Venn diagram was used to identify the common features. LASSO regression and RF modeling were implemented using the R packages ‘glmnet’ and ‘randomForest’, respectively.

Following feature acquisition, different machine learning methods, including support vector machine (SVM), RF, eXtreme Gradient Boosting (XGBoost), and logistic regression (LR) algorithms, were employed to create a model that could differentiate between cancer and noncancer individuals. The R packages ‘e1071’, ‘randomForest’, ‘xgboost’, and ‘caTools’ were utilized to implement SVM, RF, XGBoost, and LR, respectively. The models were trained with hyper-parameters tuned by 5-fold cross-validations. The final models were validated on the test set. To address potential biases in the optimization process that might arise from the specific splits employed, we replicated the above-described procedure five times, each time using a distinct splitting seed. This approach yielded five alternative optimized models to evaluate the initial model to ensure robustness. Ultimately, the efficacy of the four models was evaluated in test sets by AUROC, area under the precision–recall curve (AUPRC), sensitivity, specificity, accuracy, precision, recall and F1 score.

## Results

### Study overview

**Figure 1:**
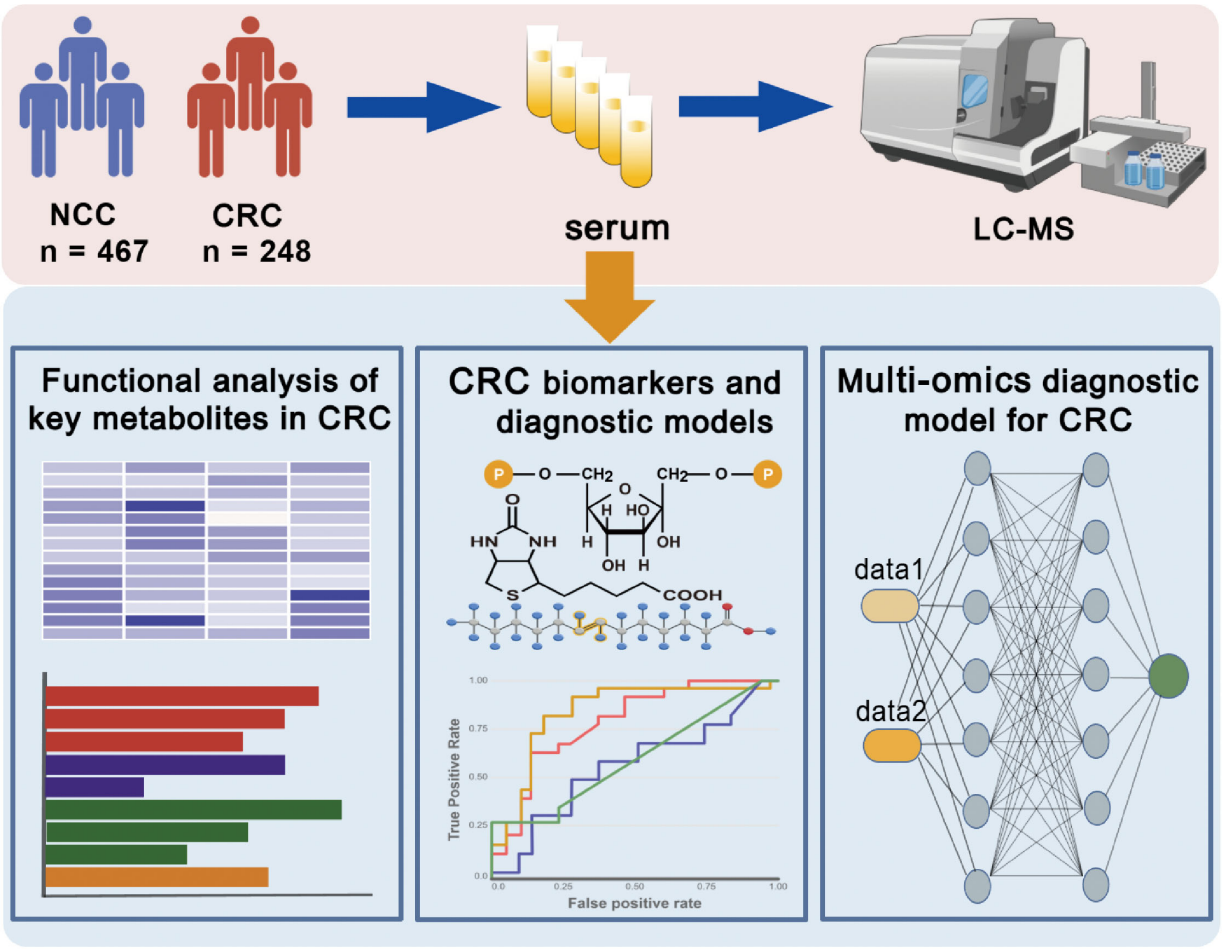
Overview of the research design. Serum samples from CRC patients and NCC individuals were collected and subjected to untargeted metabolomics. Machine learning algorithms were used to determine diagnostic biomarkers for CRC. A multi-omics model based on metabolomics was constructed to enhance the performance of the diagnostic model. This figure was created with BioGDP.com (https://BioGDP.com).

The overall workflow of this study and participant recruitment information are illustrated in **Figure 1**. A total of 715 participants were included in this study, comprising 248 CRC patients and 467 NCCs. The baseline characteristics of the study participants were summarized in **Table 1**. No statistical differences in gender, age, and BMI among the two groups (p > 0.05). The patients consisted of 50 stage I, 71 stage II, 82 stage III, 33 stage IV, and 12 unidentified stage (**Table 1**). Serum samples were collected and subjected to untargeted metabolomic profiling using LC-MS. Based on rigorous quality control and statistical filtering, differential metabolites between CRC patients and noncancer controls (NCCs) were identified and functionally annotated through pathway enrichment analysis. To assess their diagnostic potential, we applied multiple machine learning algorithms to construct predictive models using a selected panel of metabolites. In a subset of 197 participants, cfDNA methylation data were further integrated to evaluate the added value of a multi-omics classification strategy.

### Identification of differential serum metabolites in CRC

To characterize metabolic reprogramming associated with colorectal cancer, untargeted serum metabolomic profiling was performed on samples from CRC patients and NCCs. Following raw data conversion to mzXML format, preprocessing was carried out using XCMS, including peak detection, retention time correction, outlier removal, and imputation of missing values. A total of 4,030 ion features were identified in serum positive electrospray ionization (ESI+), while 674 features were identified in serum negative electrospray ionization (ESI−). Additionally, putative features were identified based on the Human Metabolome Database (HMDB) and KEGG, resulting in 2,793 annotated metabolites in ESI+ and 407 in ESI− mode.

**Figure 2:**
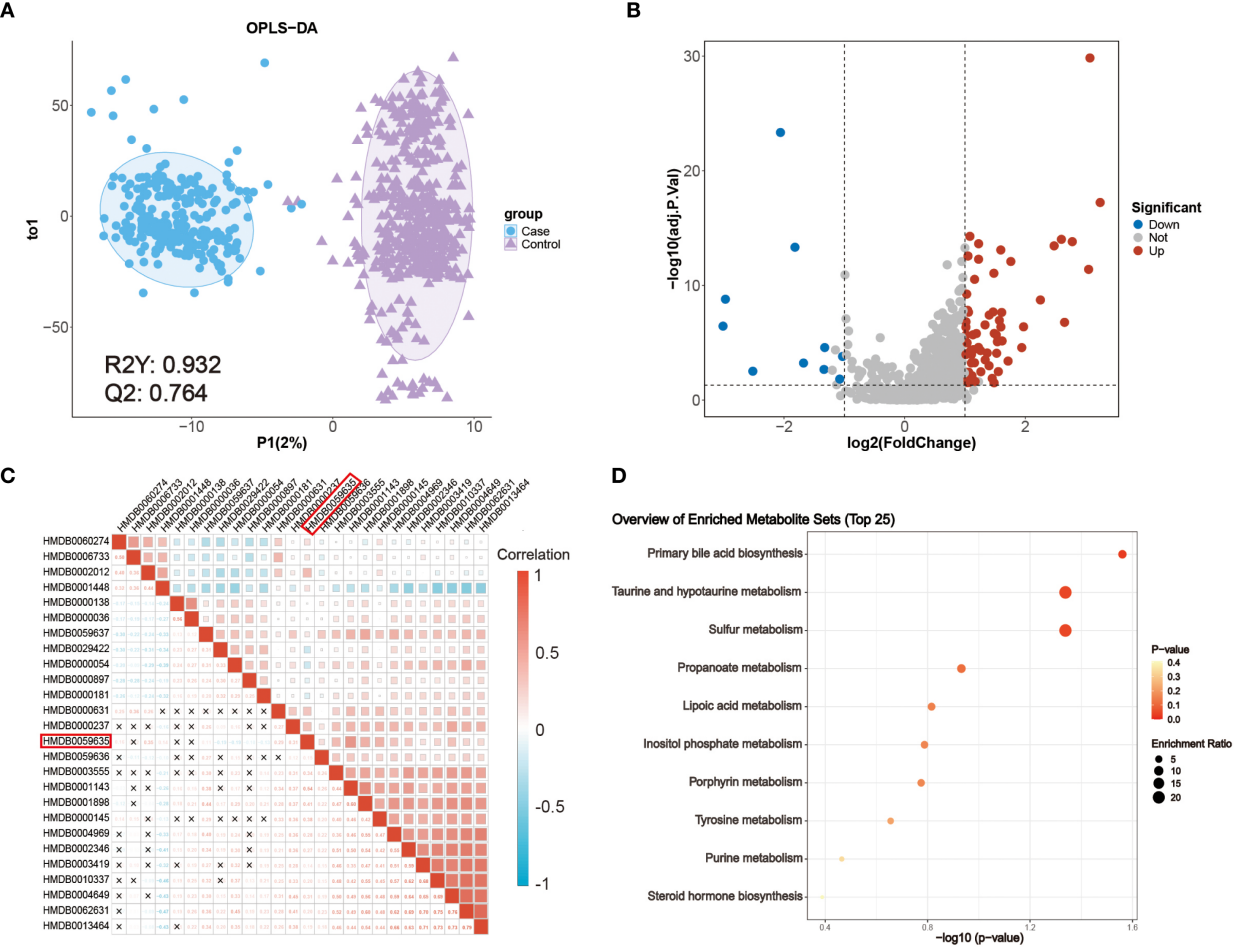
Metabolic characteristics of patients with CRC. **(A)** OPLS-DA score plot of metabolomic data from CRC (blue) and NCC (purple) samples. **(B)** Volcano plot of the detected metabolites in the serum of patients with CRC patients and NCC. Significantly differentially abundant metabolites are colored in red (upregulated) and blue (downregulated), and the others are colored in gray. A two-sided Wilcoxon rank sum test followed by the Benjamini–Hochberg (BH) multiple comparisons test was performed with FDR < 0.05, log2FC > 1 or log2FC < -1, and VIP > 1. **(C)** Correlations between 26 differentially abundant metabolites. The color represents Spearman’s correlation. Red indicates a positive correlation, and blue indicates a negative correlation. ‘×’ indicates an insignificant correlation. **(D)** KEGG metabolic pathways enriched with significantly differentially abundant metabolites between patients with CRC patients and NCC.

Univariate analysis using the Wilcoxon rank-sum test and fold change (FC) filtering was performed across all identified metabolites. In parallel, multivariate modeling with orthogonal partial least squares discriminant analysis (OPLS-DA) revealed clear separation between CRC and NCC samples (**Figure 2A**). The OPLS-DA model demonstrated strong performance with R² = 0.932 and Q² = 0.764, and permutation testing confirmed the absence of overfitting (**Supplementary Figure 1**). These results indicated robust global metabolic differences between the two groups.

A total of 75 metabolites were significantly altered in CRC (FDR < 0.05 and VIP > 1) (**Supplementary Table 1**), among which 65 were upregulated (log_2_FC > 1) and 10 were downregulated (log_2_FC < –1), as shown in the volcano plot (**Figure 2B**). Metabolite classification includes benzenoids: 8, homogeneous non-metal compounds: 2, lipids and lipid-like molecules: 20, nucleosides, nucleotides, and analogues: 4, organic acids and derivatives: 10, organic nitrogen compounds: 2, organic oxygen compounds: 5, Organic Polymers: 1, Organic salts: 1, organoheterocyclic compounds: 16, Phenylpropanoids and polyketides: 5. To identify cancer-specific endogenous markers, we excluded exogenous metabolites, such as dietary components and drug metabolites, resulting in a refined panel of 26 differentially abundant endogenous metabolites (**Supplementary Table 2**).

To explore their interrelationships, Spearman correlation analysis was performed (**Figure 2C**), revealing extensive co-regulation among several metabolites. Notably, lipoyl-AMP (HMDB0059635), a precursor involved in lysine lipoylation, a rare but evolutionarily conserved post-translational modification, was significantly upregulated. In mammals, only four core metabolic enzymes undergo lipoylation, and dysregulation of these enzymes has been implicated in various metabolic disorders. While the role of lipoyl-AMP in cancer remains unclear, its involvement in central energy pathways highlights its potential clinical relevance (22).

Pathway enrichment analysis based on KEGG annotations further revealed that several dysregulated metabolites were enriched in biologically significant pathways, including primary bile acid biosynthesis and taurine and hypotaurine metabolism (**Figure 2D**). These pathways are known to be modulated by the intestinal microbiota and play pivotal roles in CRC pathogenesis (23). In particular, taurocholic acid and its derivatives may alter microbial composition and promote the production of genotoxic substances such as hydrogen sulfide and deoxycholic acid (24). These findings underscore the close interplay between host metabolism and microbiota-derived metabolites in colorectal cancer development and support the translational value of these metabolic alterations as potential diagnostic biomarkers.

### Metabolomic biomarker panels enable CRC patients diagnosis

To optimize the diagnostic utility of serum metabolites, we applied feature selection methods to reduce redundancy among the 26 differentially abundant metabolites. Random Forest (RF) importance ranking identified 11 top-ranked metabolites (**Supplementary Figure 2A**), while Least Absolute Shrinkage and Selection Operator (LASSO) regression identified 21 metabolites with minimal binomial deviance (**Supplementary Figure 2B**). The intersection of both methods yielded 10 candidate biomarkers for model construction (**Supplementary Figure 2C**), including sulfate, ubiquinone-1, deoxycholic acid glycine conjugate, demethylphylloquinone, 3-(3,5-diiodo-4-hydroxyphenyl)lactate, vitamin K1, O-decanoyl-L-carnitine, sedoheptulose 1,7-bisphosphate, CE(22:6(4Z,7Z,10Z,13Z,16Z,19Z)), and lipoyl-AMP (**Supplementary Table 3**).

**Figure 3:**
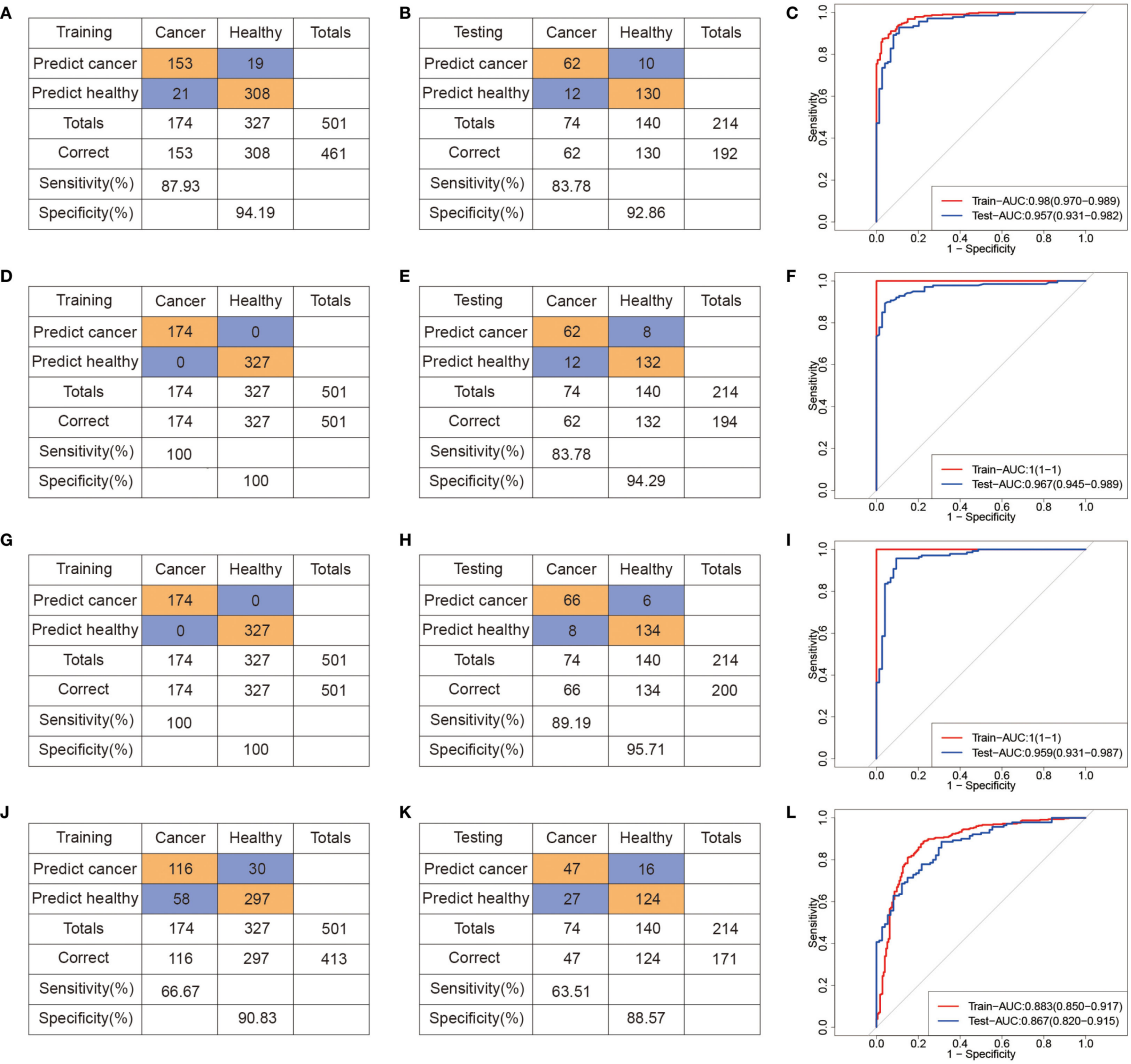
Machine learning-derived diagnostic model based on serum metabolome for CRC diagnosis. **(A–C)** Confusion tables and ROC curves of the SVM diagnostic model in the training **(A)** and testing **(B)** datasets. **(D–F)** Confusion tables and ROC curves of the RF diagnostic model in the training **(D)** and testing **(E)** datasets. **(G–I)** Confusion tables and ROC curves of the XGBoost diagnostic model in the training **(G)** and testing **(H)** datasets. **(J–L)** Confusion tables and ROC curves of the LR diagnostic model in the training **(J)** and testing **(L)** datasets.

To evaluate the diagnostic performance of these metabolites, we constructed binary classification models using both linear (logistic regression, LR) and nonlinear algorithms, including support vector machine (SVM), RF, and eXtreme Gradient Boosting (XGBoost). All samples were randomly split into a training set (70%) and a test set (30%), and the hyperparameters were selected based on tenfold cross-validation on the training set. Evaluation metrics included area under the receiver operating characteristic curve (AUROC), sensitivity, specificity, and overall accuracy (**Figure 3**). Compared to SVM (**Figures 3A–C**) and LR (**Figures 3J–L**) models, the RF (**Figures 3D–F**) and XGBoost (**Figures 3G–I**) models achieved better predictive performance. In the test cohort (74 CRC, 140 NCC), the RF model achieved robust performance, with an AUROC of 0.97, sensitivity of 83.78%, and specificity of 94.29% (**Figures 3E, F**). Similarly, the XGBoost model achieved an AUROC of 0.96 in the test set, with 89.19% sensitivity and 95.71% specificity (**Figures 3H, I**). These results demonstrate that the selected serum metabolite panel enables accurate discrimination between CRC patients and healthy controls, supporting its potential for noninvasive clinical screening applications. The results of 5 models, obtained through setting different seeds, are consistent with our initial model, which we ensure the robustness of the model (**Supplementary Table 4**).

In addition, we conducted additional analysis to evaluate whether the model distinguishes Stage I CRC from controls. The AUROC of SVM, RF, XGBoost and LR models is 0.84 (95% CI: 0.73,0.95), 0.87 (95% CI: 0.80, 0.94), 0.95 (95% CI: 0.88, 1.00) and 0.79 (95% CI: 0.66, 0.93), respectively, with low sensitivity and AUPRC (**Supplementary Figure 3**). Because of the number of Stage I CRC cases in our current dataset is limited, which may introduce variability and limit the generalizability of these findings. These results should be provided as preliminary evidence of the model’s potential in early-stage detection.

### Develop a multimodal model based on metabolomics data to enhance predictive performance

**Figure 4:**
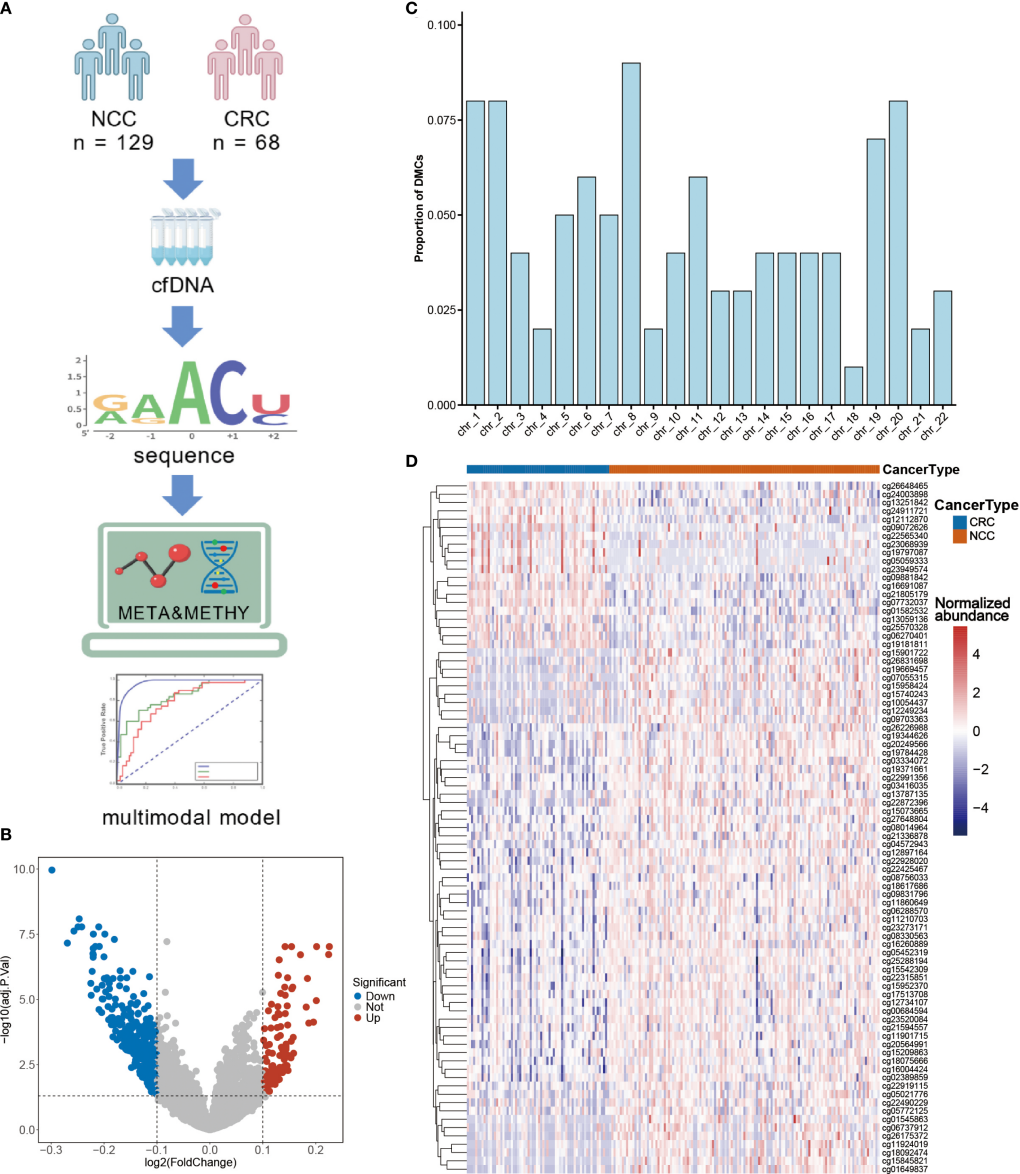
CRC-specific differential methylation sites as diagnostic biomarkers. **(A)** Cell-free DNA (cfDNA) was isolated from serum samples to perform methylation profiling, enabling the identification of differentially methylated sites. Subsequently, epigenetic markers and previously characterized differential metabolites were integrated to develop multi-omics diagnostic models. **(B)** Volcano plot of the methylation sites in the serum of CRC patients and NCC. **(C)** Proportions of differential CpG sites in different chromosomes. **(D)** Unsupervised hierarchical clustering of 83 methylation markers in promoter regions. Figure A was created with BioGDP.com (https://BioGDP.com).

To investigate whether DNA methylation features could complement metabolomic biomarkers and further improve diagnostic accuracy, we performed targeted cfDNA methylation profiling in a subset of participants (CRC, n = 68; NCC, n = 129) (**Figure 4A**, **Table 2**). Among the 3,816 high-quality CpG probes retained after preprocessing, 426 sites (11.16%) were found to be differentially methylated between CRC patients and controls (FDR < 0.05), including 110 hypermethylated (log_2_FC > 0.1) and 316 hypomethylated sites (log_2_FC < –0.1) (**Figure 4B**, **Supplementary Table 5**). Chromosomal distribution analysis revealed enrichment of differentially methylated CpG sites (DMCs) on chromosome 8, while relatively few were observed on chromosome 18 (**Figure 4C**). Given the regulatory importance of promoter methylation in gene expression, we focused our downstream analysis on DMCs located within promoter regions (**Supplementary Table 6**).

**Table 2:** Baseline characteristics of participants in methylation testing.

Characteristic	ALL	CRC	NCC	*P* value
	N=197	N=68	N=129	
Gender, No. (%)				0.077
female	85 (43.1%)	23 (33.8%)	62 (48.1%)	
male	112 (56.9%)	45 (66.2%)	67 (51.9%)	
Age, Mean (SD)	59.1 (10.7)	60.9 (11.5)	58.2 (10.1)	0.107
BMI (kg/m^2^)	23.0 (2.13)	23.1 (2.20)	23.0 (2.10)	0.778
TNM stage	NA	I: 12	NA	
		II: 21		
		III: 30		
		IV: 5		

**Figure 5:**
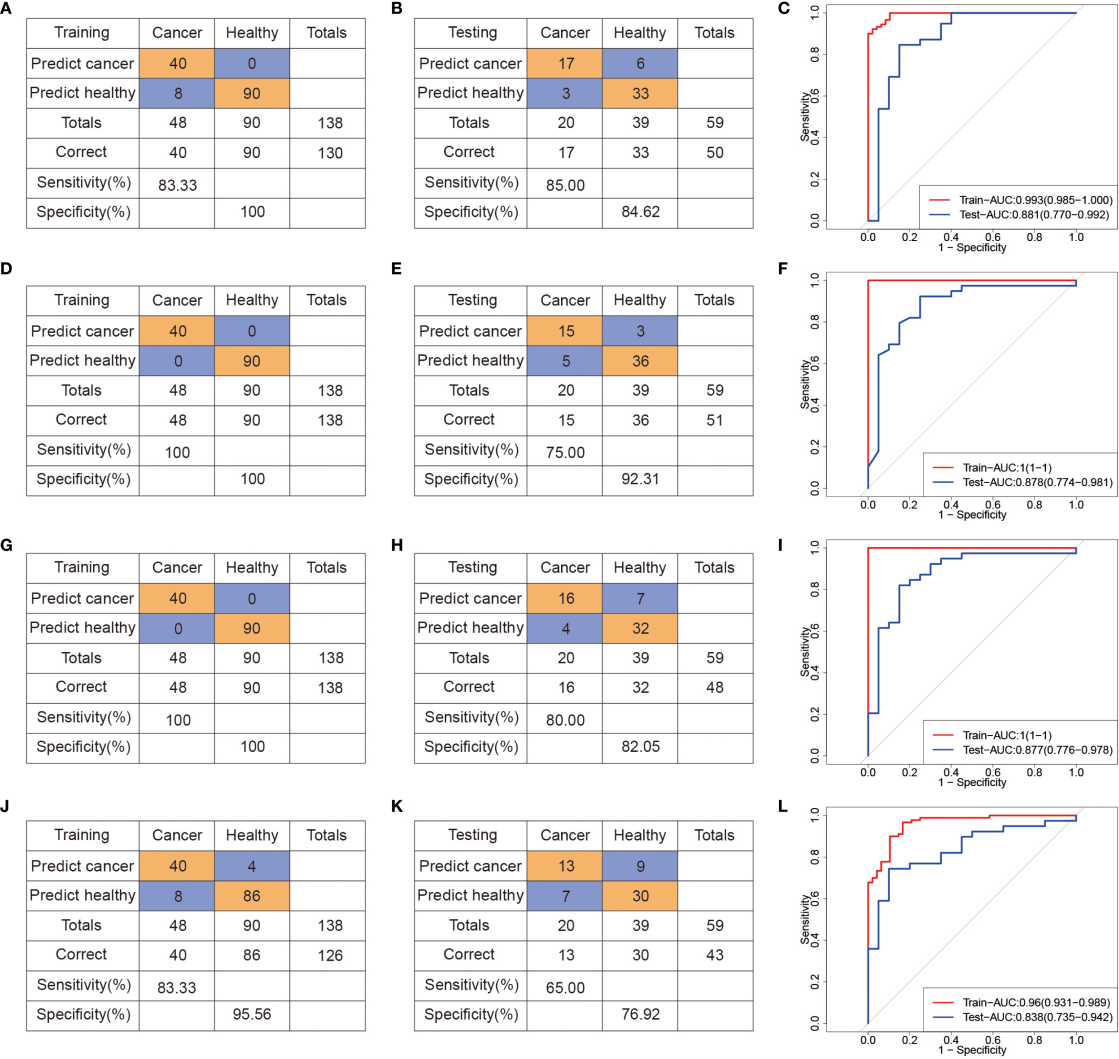
Machine learning-derived diagnostic model based on differential methylation sites for GC diagnosis. **(A-C)** Confusion tables and ROC curves of the SVM diagnostic model in the training **(A)** and testing **(B)** datasets. **(D–F)** Confusion tables and ROC curves of the RF diagnostic model in the training **(D)** and testing **(E)** datasets. **(G–I)** Confusion tables and ROC curves of the XGBoost diagnostic model in the training **(G)** and testing **(H)** datasets. **(J–L)** Confusion tables and ROC curves of the LR diagnostic model in the training **(J)** and testing **(L)** datasets.

Unsupervised hierarchical clustering of these promoter DMCs effectively discriminated CRC patients from controls (**Figure 4D**), suggesting their potential diagnostic utility. To identify a robust diagnostic methylation panel, we applied RF and LASSO regression to the training cohort (n = 138), resulting in the selection of 11 high-priority DMCs (**Supplementary Figures 2D-F**, **Supplementary Table 7**). Using these 11 features, we built classification models with SVM, RF, XGBoost, and LR algorithms (**Figure 5**). In the training cohort (48CRC, 90NCC), the sensitivity of SVM and LR models is 83.33%, with AUROC values of 0.993 and 0.96, respectively (**Figures 5A, C, J, L**). However, both RF and XGBoost models achieved 100% sensitivity and excellent AUROC values (**Figures 5D, G, F, I**). In the independent test cohort (20 CRC and 39 non-cancer controls), the SVM model achieved the highest sensitivity, reaching 85% (**Figure 5B**). Although the sensitivity of the RF and XGBoost models decreased to 75% and 80%, respectively, both maintained high specificity (**Figures 5E, H**). “In contrast, the Logistic Regression (LR) model demonstrated both sensitivity and specificity below 80% (**Figure 5K**). These results confirm the diagnostic relevance of cfDNA methylation markers but also highlight limitations in sensitivity, likely due to the smaller sample size and biological heterogeneity.

**Figure 6:**
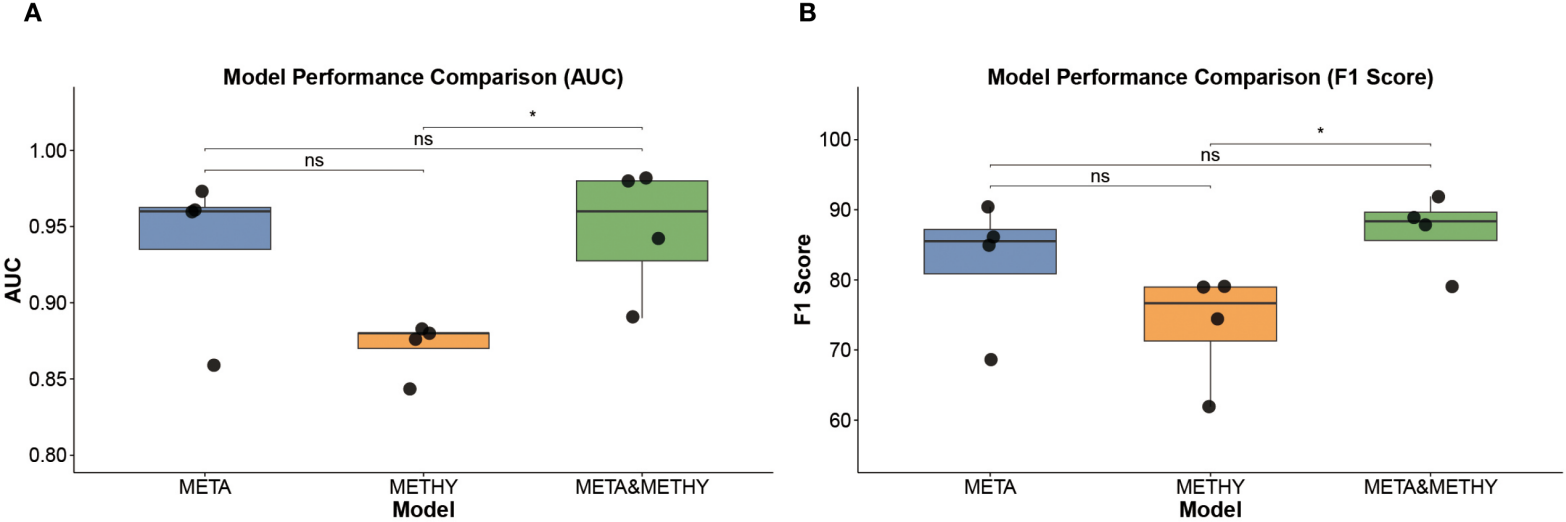
Model performance comparison. **(A)** AUROC values of META, METHY, and META&METHY models. **(B)** F1 scores of META, METHY, and META&METHY models. Wilcox test was used in **A**, **B**, * *p*<0.005, ns *p* > 0.05.

To evaluate the potential benefit of combining modalities, we next integrated the selected 10 serum metabolites with the 11 methylation markers to construct a multi-omics classifier, denoted as META&METHY. For benchmarking, we also retained the metabolomics-only (META) and methylation-only (METHY) models. Using four machine learning algorithms, RF, SVM, XGBoost, and LR, we systematically compared the performance of single-omics and multi-omics classifiers. As shown in **Figure 6A** and **Supplementary Table 7**, the META&METHY model achieved the highest diagnostic performance, with AUROCs of 0.98, AUPRC of 0.97 and 100% sensitivity and precision in the test set when using either the RF or XGBoost classifiers. F1 score comparisons further demonstrated the superiority of the META&METHY model over both the META and METHY models (**Figure 6B**). Notably, the metabolomics-only models consistently outperformed the methylation-only models, with AUROCs ranging from 0.87 to 0.97 versus 0.84 to 0.88, respectively (**Supplementary Table 8**). These findings suggest that while methylation features provide valuable supplementary information, serum metabolites are the dominant contributors to model performance.

In summary, the integration of cfDNA methylation with serum metabolomics modestly enhances predictive power, particularly in terms of sensitivity and classification confidence. However, the high performance of metabolomics-based models alone underscores their potential as a standalone, noninvasive diagnostic tool for colorectal cancer. Future studies with expanded cohorts are warranted to further validate and optimize this multi-omics approach for clinical application.

## Discussion

In this study, we conducted a large-scale investigation involving 715 participants to explore serum metabolic alterations associated with colorectal cancer (CRC) and to identify noninvasive biomarkers for early detection. Using untargeted metabolomics coupled with high-resolution LC-MS, we identified 75 distinct metabolic signatures that differentiated CRC patients from noncancer controls (NCC). Through a comparative analysis with the metabolomic biomarkers reported in Yu’s 2024 study (5), we noted that, despite the identification of distinct metabolite entities in the two studies, both cohorts exhibited analogous alterations in key metabolic categories, including sulfates, histidine derivatives, bile acids, and lipids. These observations underscore the existence of shared metabolic reprogramming pathways integral to the pathogenesis of colorectal cancer, thereby providing a robust foundation for subsequent mechanistic investigations.

Feature selection and machine learning-based modeling enabled the construction of a robust diagnostic classifier based on 10 key metabolites. These metabolites play an important role in the occurrence and development of cancer. Ubiquinone plays a pivotal role in modulating mitochondrial oxidative phosphorylation (OXPHOS) activation and reactive oxygen species (ROS) generation. It can facilitate the progression of CRC by activating the PI3K/AKT signaling pathway in a ROS-dependent manner (25, 26). Demethylphylloquinone is a late biosynthetic intermediate of vitamin K1 (27). Recent research has unveiled novel functions of vitamin K within cancer cells, encompassing the activation of the steroid and xenobiotic receptor (SXR), as well as the modulation of oxidative stress responses, apoptotic pathways, and autophagic processes (28). Lipoyl-AMP is essential for the lipoylation reaction which is a rare but highly conserved post-translational modification of lysine. Only four polymer-metabolizing enzymes are known to undergo lipid acylation in mammals, and these proteins are major constituents of core metabolism. Dysregulation of these proteins is associated with a variety of human metabolic disorders. Thus lipoyl-AMP is strongly implicated in the maintenance of health and the development of disease, although the exact mechanisms remain to be elucidated (29).

In a subset of participants, we further incorporated cfDNA methylation data to build an integrated multi-omics diagnostic model. While both single-omics and multi-omics models demonstrated high diagnostic accuracy, the metabolomics-based models consistently outperformed methylation-only models, highlighting the central role of metabolic alterations in CRC detection.

CRC remains a major global health challenge, and patient outcomes are strongly influenced by the stage at diagnosis. Traditional diagnostic methods such as colonoscopy, although accurate, are invasive and often poorly accepted by patients. Liquid biopsy has emerged as a promising alternative, and recent studies have focused on circulating nucleic acids and proteins as diagnostic biomarkers (30–33). However, metabolomics remains underutilized despite its unique advantages. Our study reinforces the value of serum metabolites as clinically accessible biomarkers for CRC. Compared to proteomic or genomic approaches, metabolomics offers several advantages, including relatively low cost, simpler sample preparation, and greater patient compliance due to its noninvasive nature.

To translate these metabolomic insights into practical diagnostic tools, we applied machine learning algorithms, RF, XGBoost, SVM, and LR, to build predictive models. The combination of RF and LASSO was used to reduce feature dimensionality and eliminate noise. Among the four classifiers tested, RF and XGBoost consistently exhibited the highest performance across training and test datasets, achieving AUROCs up to 0.97 in independent validation. These results support the utility of metabolomics for developing accurate, reproducible, and interpretable models for CRC detection.

In addition to evaluating the metabolomics-derived models, we explored whether cfDNA methylation data could provide complementary diagnostic information. A panel of 11 differentially methylated CpG sites was identified, and models based solely on methylation features showed moderate performance. However, when integrated with metabolite features in a multi-omics model (META&METHY), predictive performance was further enhanced, achieving an AUROC of 0.98 (95% CI: 0.95, 1.00) and high precision in the test set. While these findings suggest that DNA methylation may contribute additional information, our data clearly demonstrate that serum metabolomics remains the dominant contributor to classification accuracy, and may suffice as a standalone diagnostic modality in many clinical scenarios.

Despite the promising results, we acknowledge several limitations. First, although this study includes a relatively large overall cohort, the subset used for multi-omics analysis remains limited. We fully recognize that reliance on a single clinical site may limit the generalizability of our findings due to inherent demographic, geographic, or procedural variations. We will recruit participants from different geographical and socio-economic backgrounds, standardize metabolomics analysis plans for different locations to minimize technical differences, and perform stratified analysis to evaluate whether the observed metabolic changes are consistent across subgroups (e.g. by age, gender, or cancer stage). By conducting this multi-center analysis, we anticipate strengthening the evidence for the generalizability of our observations and addressing potential sampling biases inherent in the initial single-center design. Second, to enable clinical application, future work will focus on functional studies (e.g., cell/animal models) to elucidate the roles of poorly characterized metabolites in CRC. Targeted quantification of key metabolites using isotope-labeled standards in multicenter cohorts, establishing clinical thresholds and reference ranges. Validate assay performance (sensitivity, specificity) in independent cohorts. Assess cost-effectiveness and scalability compared to existing CRC screening methods (e.g., colonoscopy). Third, while our study incorporated both metabolomic and methylation data for diagnostic modeling, their mechanistic interactions in CRC biology were only briefly explored and merit more in-depth investigation in future studies. Elucidating the crosstalk between these molecular layers may offer deeper insights into tumor biology and identify therapeutic targets. Fourth, although our cohort reflects clinical patients undergoing diagnostic evaluation for CRC, its CRC prevalence (34.7%) exceeds that of asymptomatic screening populations (<1%). Therefore, future validation in large asymptomatic cohorts is required to confirm its utility in screening settings. Lastly, potential confounding factors such as diet, medication, and lifestyle were not fully controlled and warrant further investigation.

## Conclusion

In summary, we conducted a comprehensive serum metabolomics study to identify diagnostic biomarkers for colorectal cancer. Our findings demonstrate significant metabolic dysregulation in CRC patients and highlight a panel of metabolites with strong diagnostic potential. Machine learning-based models built on these features exhibited high accuracy, sensitivity, and specificity. The integration of DNA methylation data provided additional value, although the metabolomics signature alone retained superior performance. These results support the clinical utility of metabolomics as a noninvasive, cost-effective liquid biopsy tool for CRC screening and lay the foundation for future translational applications through targeted validation in large, multicenter studies.

## Data Availability

The original contributions presented in the study are included in the article/**Supplementary Material**, further inquiries can be directed to the corresponding author/s.

## References

[1] Bray F Laversanne M Sung H Ferlay J Siegel RL Soerjomataram I. Global cancer statistics 2022: GLOBOCAN estimates of incidence and mortality worldwide for 36 cancers in 185 countries. CA Cancer J Clin. (2024) 74:229–63. doi: 10.3322/caac.21834, PMID: 38572751

[2] Pinsky PF. Principles of cancer screening. Surg Clin North Am. (2015) 95:953–66. doi: 10.1016/j.suc.2015.05.009, PMID: 26315516 PMC4555845

[3] Xia C Basu P Kramer BS Li H Qu C Yu XQ. Cancer screening in China: a steep road from evidence to implementation. Lancet Public Health. (2023) 8:e996–e1005. doi: 10.1016/S2468-2667(23)00186-X, PMID: 38000379 PMC10665203

[4] Zhang A Sun H Yan G Wang P Han Y Wang X. Metabolomics in diagnosis and biomarker discovery of colorectal cancer. Cancer Lett. (2014) 345:17–20. doi: 10.1016/j.canlet.2013.11.011, PMID: 24333717

[5] Sun Y Zhang X Hang D Lau HC Du J Liu C. Integrative plasma and fecal metabolomics identify functional metabolites in adenoma-colorectal cancer progression and as early diagnostic biomarkers. Cancer Cell. (2024) 42:1386–400.e8. doi: 10.1016/j.ccell.2024.07.005, PMID: 39137727

[6] McCullough ML ML Hodge RA Campbell PT Stevens VL Wang Y. Pre-diagnostic circulating metabolites and colorectal cancer risk in the cancer prevention study-II nutrition cohort. Metabolites. (2021) 11:156. doi: 10.3390/metabo11030156, PMID: 33803340 PMC8000483

[7] Vidman L Zheng R Bodén S Ribbenstedt A Gunter MJ Palmqvist R. Untargeted plasma metabolomics and risk of colorectal cancer-an analysis nested within a large-scale prospective cohort. Cancer Metab. (2023) 11:17. doi: 10.1186/s40170-023-00319-x, PMID: 37849011 PMC10583301

[8] Xie Z Zhu R Huang X Yao F Jin S Huang Q. Metabolomic analysis of gut metabolites in patients with colorectal cancer: Association with disease development and outcome. Oncol Lett. (2023) 26:358. doi: 10.3892/ol.2023.13944, PMID: 37545617 PMC10398631

[9] Zhang SL Cheng LS Zhang ZY Sun HT Li JJ. Untangling determinants of gut microbiota and tumor immunologic status through a multi-omics approach in colorectal cancer. Pharmacol Res. (2023) 188:106633. doi: 10.1016/j.phrs.2022.106633, PMID: 36574857

[10] Chen F Dai X Zhou CC Li KX Zhang YJ Lou XY. Integrated analysis of the faecal metagenome and serum metabolome reveals the role of gut microbiome-associated metabolites in the detection of colorectal cancer and adenoma. Gut. (2022) 71:1315–25. doi: 10.1136/gutjnl-2020-323476, PMID: 34462336 PMC9185821

[11] Yang Y Wang Z Li X Lv J Zhong R Gao S. Profiling the metabolic disorder and detection of colorectal cancer based on targeted amino acids metabolomics. J Transl Med. (2023) 21:824. doi: 10.1186/s12967-023-04604-7, PMID: 37978537 PMC10655464

[12] Troisi J Tafuro M Lombardi M Scala G Richards SM Symes SJK. A metabolomics-based screening proposal for colorectal cancer. Metabolites. (2022) 12:110. doi: 10.3390/metabo12020110, PMID: 35208185 PMC8878838

[13] Yi Y Wang J Liang C Ren C Lian X Han C. LC-MS-based serum metabolomics analysis for the screening and monitoring of colorectal cancer. Front Oncol. (2023) 13:1173424. doi: 10.3389/fonc.2023.1173424, PMID: 37448516 PMC10338013

[14] Xu Z Li W Dong X Chen Y Zhang D Wang J. Precision medicine in colorectal cancer: Leveraging multi-omics, spatial omics, and artificial intelligence. Clin Chim Acta. (2024) 559:119686. doi: 10.1016/j.cca.2024.119686, PMID: 38663471

[15] Adusumilli R Mallick P. Data conversion with proteoWizard msConvert. Methods Mol Biol. (2017) 1550:339–68. doi: 10.1007/978-1-4939-6747-623, PMID: 28188540

[16] Domingo-Almenara X Siuzdak G. Metabolomics data processing using XCMS. Methods Mol Biol. (2020) 2104:11–24. doi: 10.1007/978-1-0716-0239-32 31953810

[17] Wishart DS Tzur D Knox C Eisner R Guo AC Young N. HMDB: the human metabolome database. Nucleic Acids Res. (2007) 35:D521–6. doi: 10.1093/nar/gkl923, PMID: 17202168 PMC1899095

[18] Kanehisa M Goto S. KEGG: kyoto encyclopedia of genes and genomes. Nucleic Acids Res. (2000) 28:27–30. doi: 10.1093/nar/28.1.27, PMID: 10592173 PMC102409

[19] Shen X Wu S Liang L Chen S Contrepois K Zhu ZJ. metID: an R package for automatable compound annotation for LC-MS-based data. Bioinformatics. (2022) 38:568–9. doi: 10.1093/bioinformatics/btab583, PMID: 34432001 PMC8722759

[20] Shen X Wang R Xiong X Yin Y Cai Y Ma Z. Metabolic reaction network-based recursive metabolite annotation for untargeted metabolomics. Nat Commun. (2019) 10:1516. doi: 10.1038/s41467-019-09550-x, PMID: 30944337 PMC6447530

[21] Luan H Ji F Chen Y Z. statTarget: A streamlined tool for signal drift correction and interpretations of quantitative mass spectrometry-based omics data. Anal Chim Acta. (2018) 1036:66–72. doi: 10.1016/j.aca.2018.08.002, PMID: 30253838

[22] Rowland EA Snowden CK Cristea IM. Protein lipoylation: an evolutionarily conserved metabolic regulator of health and disease. Curr Opin Chem Biol. (2018) 42:76–85. doi: 10.1016/j.cbpa.2017.11.003, PMID: 29169048 PMC5965299

[23] Cong J Liu P Han Z Ying W Li C Yang Y. Bile acids modified by the intestinal microbiota promote colorectal cancer growth by suppressing CD8(+) T cell effector functions. Immunity. (2024) 57:876–89.e11. doi: 10.1016/j.immuni.2024.02.014, PMID: 38479384

[24] Ridlon JM Wolf PG Gaskins HR. Taurocholic acid metabolism by gut microbes and colon cancer. Gut Microbes. (2016) 7:201–15. doi: 10.1080/19490976.2016.1150414, PMID: 27003186 PMC4939921

[25] Liu Z Chaillou T Alves Santos E Mader T Jude B Ferreira DMS. Mitochondrial NDUFA4L2 is a novel regulator of skeletal muscle mass and force. FASEB J. (2021) 35:e22010. doi: 10.1096/fj.202100066R, PMID: 34724256

[26] Ye N Wang Y Jiang P Jiang H Ding W Zhang Z. Hypoxia-induced the upregulation of NDUFA4L2 promoted colon adenocarcinoma progression through ROS-mediated PI3K/AKT pathway. Cytotechnology. (2023) 75:461–72. doi: 10.1007/s10616-023-00590-2, PMID: 37841958 PMC10575837

[27] Fatihi A Latimer S Schmollinger S Block A Dussault PH Vermaas WF. A dedicated type II NADPH dehydrogenase performs the penultimate step in the biosynthesis of vitamin K1 in synechocystis and arabidopsis. Plant Cell. (2015) 27:1730–41. doi: 10.1105/tpc.15.00103, PMID: 26023160 PMC4498204

[28] Welsh J Bak MJ Narvaez CJ. New insights into vitamin K biology with relevance to cancer. Trends Mol Med. (2022) 28:864–81. doi: 10.1016/j.molmed.2022.07.002, PMID: 36028390 PMC9509427

[29] Shi Q Shen Q Liu Y Shi Y Huang W Wang X. Increased glucose metabolism in TAMs fuels O-GlcNAcylation of lysosomal Cathepsin B to promote cancer metastasis and chemoresistance. Cancer Cell. (2022) 40:1207–22.e10. doi: 10.1016/j.ccell.2022.08.012, PMID: 36084651

[30] Zhang Y Wang Y Zhang B Li P Zhao Y. Methods and biomarkers for early detection, prediction, and diagnosis of colorectal cancer. BioMed Pharmacother. (2023) 163:114786. doi: 10.1016/j.biopha.2023.114786, PMID: 37119736

[31] Torresan S Scordilli M Bortolot M Nardo Di P Foltran L Fumagalli A. Liquid biopsy in colorectal cancer: Onward and upward. Crit Rev Oncol Hematol. (2024) 194:104242. doi: 10.1016/j.critrevonc.2023.104242, PMID: 38128627

[32] Mei H Wen Y. MicroRNAs for diagnosis and treatment of colorectal cancer. Endocr Metab Immune Disord Drug Targets. (2021) 21:47–55. doi: 10.2174/1871530320999200818134339, PMID: 32819240

[33] Martín-García D García-Aranda M Redondo M. Biomarker identification through proteomics in colorectal cancer. Int J Mol Sci. (2024) 25:2283. doi: 10.3390/ijms25042283, PMID: 38396959 PMC10888664

