# Supplementary material for: Serum metabolomics-based diagnostic biomarkers for colorectal cancer: insights and multi-omics validation
Source: Front Endocrinol (Lausanne). 2025 Oct 27;16:1663938. doi: 10.3389/fendo.2025.1663938 (PMC12597806; doi:10.3389/fendo.2025.1663938)
Supplement: Supplementary file 1 [file DataSheet1.docx]

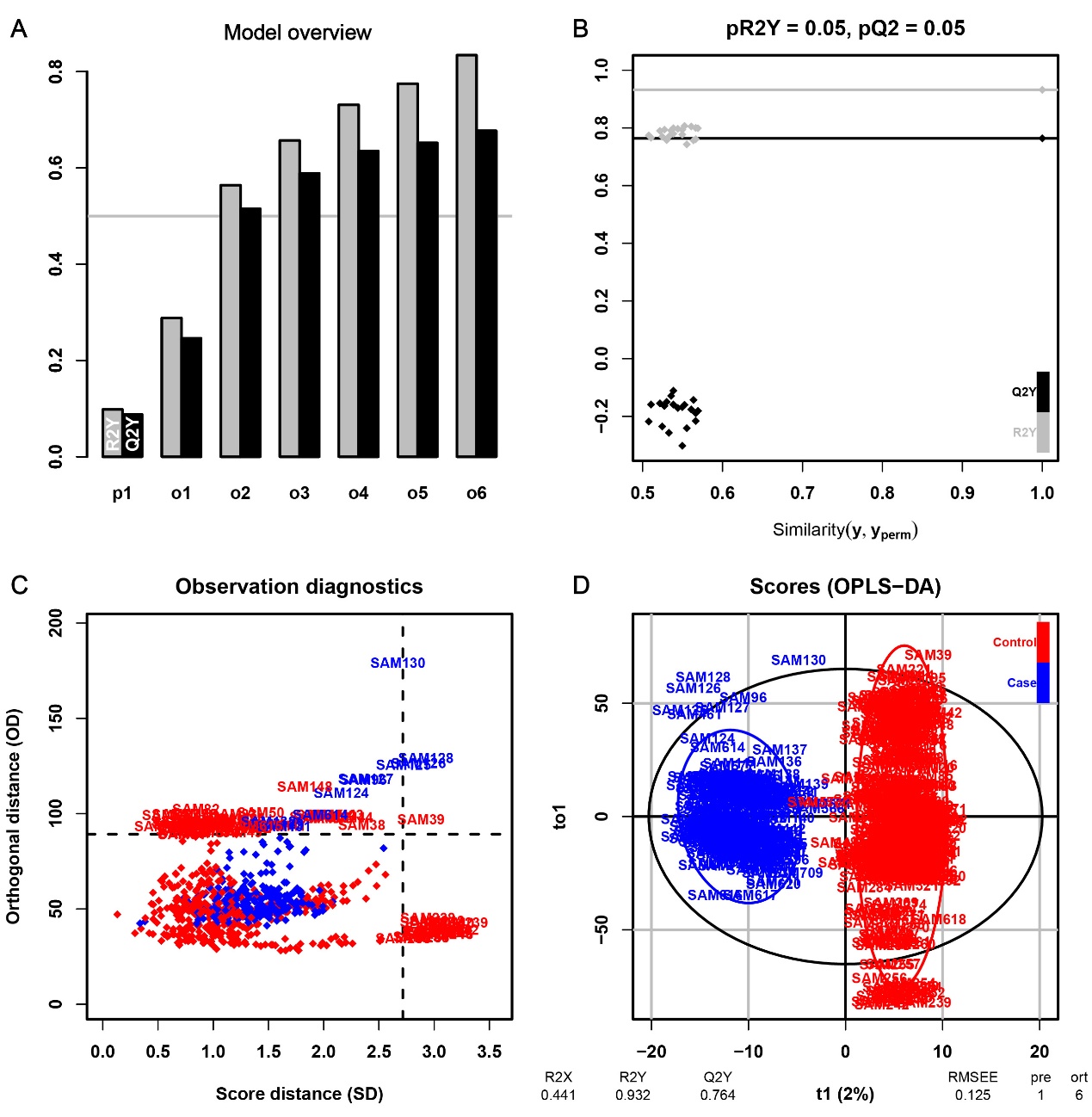


**Supplementary Fig. 1 OPLS-DA model assessment** (A) Inertia bar chart. (B) Model validation plot (permutation test). (C) Outlier diagnostic plot. (D) OPLS-DA score plot.


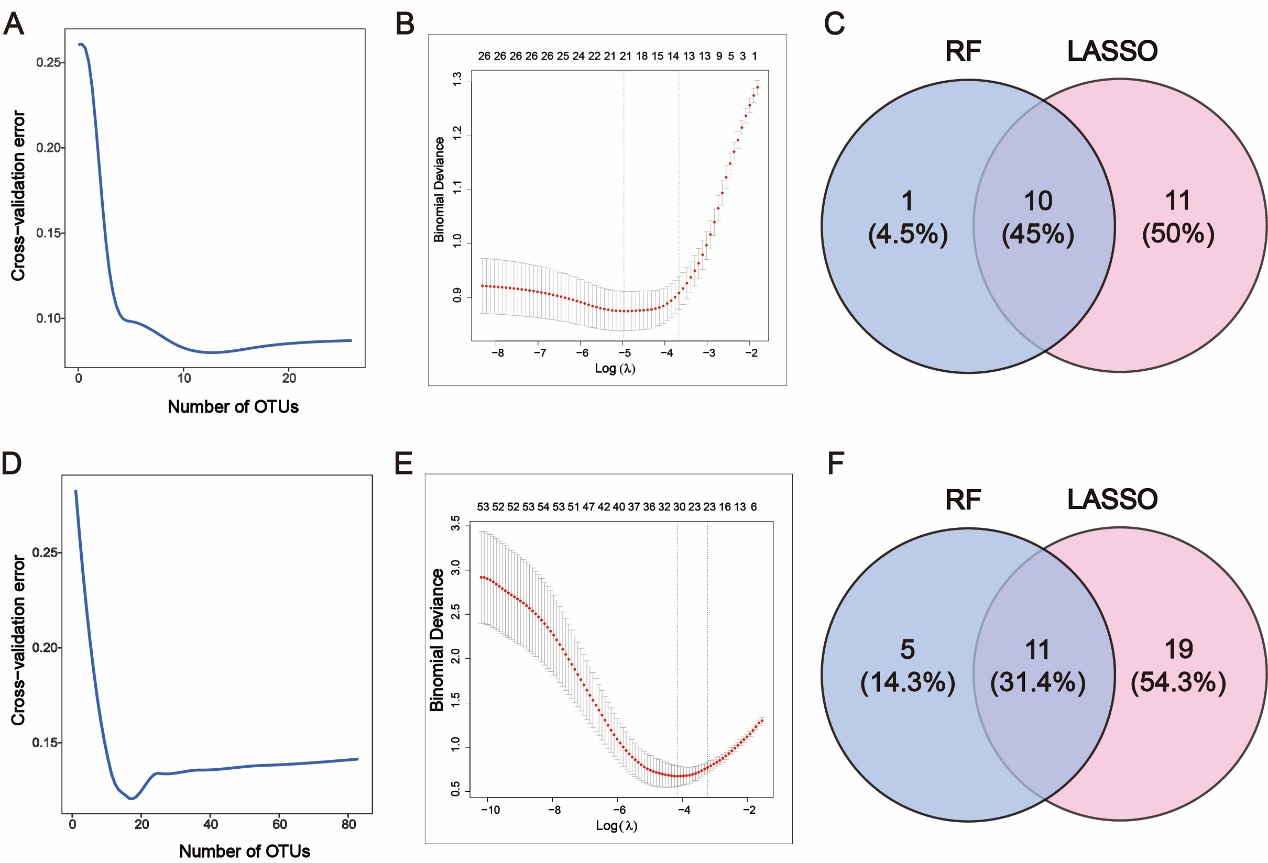


**Supplementary Fig. 2 Feature screening** (A), (D) Features of metabolic (A) and methylation (D) screening in the RF model. (B), (E) Features of metabolic (B) and methylation (E) screening in the LASSO regression model. (C), (F) Venn diagrams of differentially abundant metabolites (C) and methylation sites (F) overlapping between the RF and LASSO algorithms.


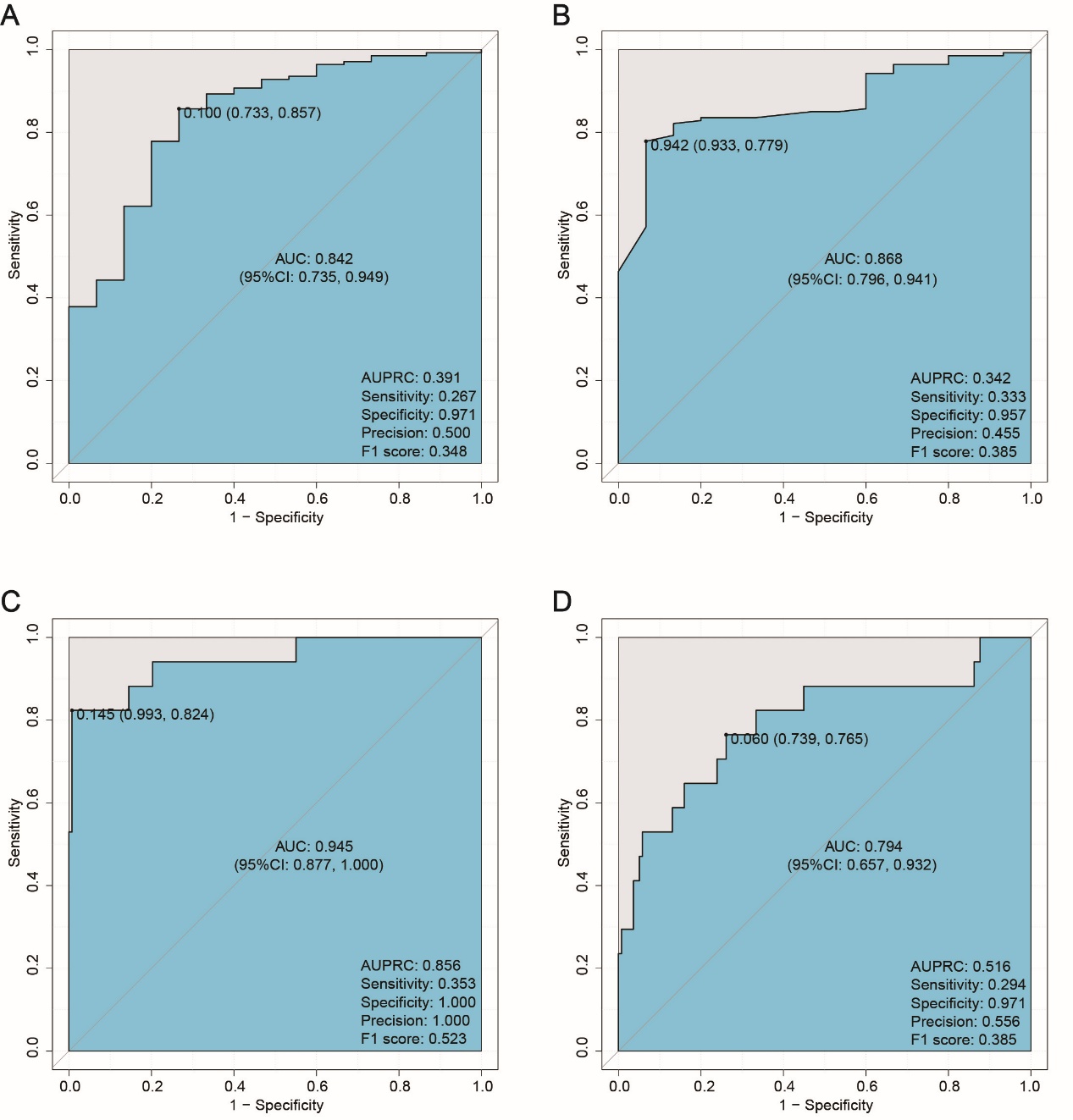


**Supplementary Fig. 3 AUROC analysis for models to discriminate stage I CRC from NC** (A)-(D) AUROC curves of the SVM (A), RF (B), XGBoosst (C) and LR (D)diagnostic model
